# The physiological functions of ascorbate in the development of cancer

**DOI:** 10.1242/dmm.052201

**Published:** 2025-04-11

**Authors:** Michalis Agathocleous

**Affiliations:** ^1^ Children's Medical Center Research Institute, University of Texas Southwestern Medical Center, Dallas, TX 75235, USA; ^2^ Department of Pediatrics, University of Texas Southwestern Medical Center, Dallas, TX 75235, USA

## Abstract

The metabolite ascorbate (vitamin C) is synthesized endogenously in most animals or, in humans and some other species, obtained from the diet. Its role in cancer development is controversial. Addition of ascorbate to cultured cells or high-dose administration in animals can inhibit growth of many cancers, but most of these effects are caused by non-physiological biochemical activities. Few experiments have tested the physiological roles of ascorbate in cancer development by depleting it in physiological settings. Ascorbate depletion inhibits the activity of ten-eleven translocation (TET) enzymes in hematopoietic and leukemia cells and accelerates myeloid leukemia development. Many clinical trials have tested ascorbate supplementation in cancers and shown little or no evidence that it has a beneficial role. I propose that depletion experiments are needed to define the cancers in which ascorbate has a physiological role, establish its cellular and molecular targets, and provide a rationale for clinical trials.

## Teaser

**Summary:** This Perspective argues that the physiological functions of ascorbate in the development of cancer must be defined using ascorbate depletion experiments. This will inform the design of clinical trials of ascorbate in cancer prevention or treatment.

## Introduction

Ascorbate is an abundant endogenous metabolite synthesized from glucose by a dedicated biosynthetic pathway ancestral to all eukaryotes (Wheeler et al., 2015). It has diverse biochemical roles, including as a major antioxidant (Frei et al., 1989; Harrison et al., 2010) and as a promoter of the activity of some iron- and α-ketoglutarate-dependent dioxygenase and copper type-II monooxygenase enzymes (Cimmino et al., 2018). Ascorbate was originally isolated as a potent reducing agent (Szent-Gyorgyi, 1928) and then identified as vitamin C (Svirbely and Szent-Gyorgyi, 1932), the dietary molecule essential to prevent or treat scurvy. Most animals do not develop scurvy when deprived of dietary ascorbate because of endogenous synthesis. However, the terminal ascorbate biosynthesis enzyme L-gulonolactone oxidase (GULO) has been lost in some species, including humans, guinea pigs, bats, and many fishes and birds, rendering them dependent on dietary ascorbate (Wheeler et al., 2015). Vitamin C is thus unusual in that it is a vitamin only for us and a few other species. This observation immediately raised the question of whether ascorbate deficiency contributes to diseases other than scurvy. Despite the fact that ascorbate deficiency is common in humans (Khaw et al., 2001; Loria et al., 2000; Schleicher et al., 2009), this question remains unresolved.

Here, I argue that, to understand the role of ascorbate in disease, it is necessary to test the effects of depletion (i.e. loss of function) *in vivo*. Loss-of-function experiments are the standard approach to test the roles of endogenous molecules. But, despite thousands of papers on ascorbate and cancer, it has not been tested whether ascorbate depletion promotes initiation of any cancer *in vivo* apart from myeloid leukemia (Agathocleous et al., 2017). Much work has tested the effects of ascorbate administration (i.e. gain of function) in humans or mice with normal baseline ascorbate levels, or the effects of ascorbate *in vitro*. However, supraphysiological ascorbate can inhibit cell growth by inducing oxidative stress, an effect distinct from its physiological antioxidant function (Halliwell, 2014; Levine et al., 2011). Despite the lack of knowledge on whether physiological ascorbate levels modulate cancer development, ascorbate administration has been tested in several cancer prevention or treatment trials, most of which have shown no effects (Frei et al., 2012; Creagan et al., 1979; Moertel et al., 1985). Ascorbate may work via distinct mechanisms to modulate the initiation or progression of different cancers. Understanding such mechanisms is essential to design trials that test whether ascorbate can help prevent or treat specific cancers.

### Ascorbate metabolism

In mammals, ascorbate is synthesized in the liver or consumed in the diet, then absorbed into the blood and taken up by cells through two dedicated high-affinity transporters, solute carrier family 23 members SLC23A1 and SLC23A2 (Padayatty and Levine, 2016). SLC23A1 is mainly expressed in epithelial tissues involved in bulk transport, including the kidney, liver or intestine, and SLC23A2 is expressed in most tissues (May, 2011). SLC23A1 and SLC23A2 concentrate ascorbate such that it can reach millimolar intracellular concentrations (Bergsten et al., 1990; Agathocleous et al., 2017; Levine et al., 2001), making it one of the most abundant intracellular molecules in organisms with sufficient synthesis or intake. Ascorbate can also be transported into cells in its oxidized form, dehydroascorbate, through GLUT (SLC2A) glucose transporters (May, 2011). This is likely to be physiologically important only in cells that do not express SLC23A1 or SLC23A2, such as erythrocytes (Tu et al., 2015), or cells in highly oxidative environments, such as activated neutrophils (Washko et al., 1993).

Ascorbate is an antioxidant (Frei et al., 1989) and promotes collagen synthesis (Myllyla et al., 1984), bone formation (Thaler et al., 2022), differentiation of several types of immune cells (Yue and Rao, 2020) and noradrenaline synthesis (Bornstein et al., 2003), among other functions. However, most cells in culture grow without ascorbate, and humans can remain on an ascorbate-deficient diet for months before serious pathologies appear (Krebs, 1953; Crandon et al., 1940). Thus, despite its ancient origin, intracellular abundance and biochemical and physiological versatility, ascorbate is not essential for cellular life. In order to define the processes that require ascorbate *in vivo*, it is necessary, just like with any other endogenous molecule, to perform loss-of-function experiments. Systemic ascorbate depletion can be achieved in animals that are *Gulo* deficient and cannot synthesize ascorbate (Maeda et al., 2000). Their ascorbate levels can be controlled by varying amounts of dietary supplementation. For example, the *Gulo*-deficient guinea pig was key in understanding that scurvy is caused by dietary ascorbate deficiency (Svirbely and Szent-Gyorgyi, 1932; Holst and Frolich, 1907). *Gulo*-knockout mice fed an ascorbate-deficient diet have ascorbate depletion, similar to *GULO-*deficient humans (Maeda et al., 2000). However, some differences in ascorbate transport mechanisms exist between species that are naturally *GULO* deficient and engineered *Gulo-*deficient mice (Montel-Hagen et al., 2008; Michels and Frei, 2013)*.* Ascorbate can also be depleted within cells by inhibiting its uptake through deletion of *Slc23a2* (Sotiriou et al., 2002) or *Slc23a1* (Corpe et al., 2010)*.* This can be done in a cell-type-specific manner without systemic depletion (Comazzetto et al., 2024; Agathocleous et al., 2017). Ascorbate has not been depleted specifically in cancer cells *in vivo* to determine its cell-autonomous roles in cancer development.

[…] despite the long history of work on ascorbate and cancer, the cellular and molecular targets of ascorbate in the development of most cancers have not been defined with rigorous loss-of-function experiments.

### Physiological ascorbate and cancer development in experimental models

Ascorbate promotes the activity of the ten-eleven translocation (TET) family enzymes in purified enzyme systems, cultured cells, animal models and humans (Blaschke et al., 2013; Chen et al., 2013; Minor et al., 2013; Yin et al., 2013; Hore et al., 2016; Agathocleous et al., 2017; Cimmino et al., 2017; Liu et al., 2016; Guan et al., 2020; He et al., 2015; Gillberg et al., 2019). TET1-3 are α-ketoglutarate-dependent dioxygenases that convert 5-methylcytosine to 5-hydroxymethylcytosine and other oxidized derivatives on DNA and RNA (Tahiliani et al., 2009; Ito et al., 2011). Thus, TET deficiency can change gene expression or impair genome stability (Lopez-Moyado et al., 2024). Inactivating *TET2* mutations are prevalent in acute myeloid leukemia (AML) (Papaemmanuil et al., 2016) and in other blood cancers. These mutations are also present in clonally expanded hematopoietic stem cells (HSCs) and blood cells of older individuals, and predispose individuals to AML development (Jaiswal and Ebert, 2019). Intracellular ascorbate is elevated in mouse and human HSCs compared to in other hematopoietic cells (Agathocleous et al., 2017). Ascorbate depletion in mice to the low levels observed in some humans reduces TET activity in HSCs and hematopoietic progenitors (Agathocleous et al., 2017). Cell-intrinsic ascorbate depletion in mice by deletion of the *Slc23a2* transporter in the context of normal circulating levels increases self-renewal of HSCs (Agathocleous et al., 2017) and of multipotent progenitors (Comazzetto et al., 2024). Systemic or cell-intrinsic ascorbate depletion in *Gulo^−/−^* mice or *Slc23a2^−/−^* hematopoietic cells, respectively, expands populations of hematopoietic cells carrying the *Flt3^ITD^* leukemogenic mutation, an effect mediated by TET2 (Agathocleous et al., 2017). Systemic ascorbate depletion promotes the development of *Tet2^+/−^* or *Tet2^−/−^* myeloid neoplasms, suggesting TET2-dependent and TET2-independent effects (Agathocleous et al., 2017; Guan et al., 2020). All effects of ascorbate depletion in *Gulo^−/−^* mice are reversed by dietary ascorbate (Agathocleous et al., 2017). Therefore, in mice, ascorbate is a physiological suppressor of HSC and progenitor self-renewal, and of myeloid neoplasm development. Ascorbate supplementation increases TET activity in blood cells of ascorbate-deficient patients with leukemia (Gillberg et al., 2019) or of *TET2* germline mutation carriers (Taira et al., 2023), suggesting that ascorbate is also a physiological TET regulator in humans.

In other cancers, B16-F10 melanoma cells (Campbell et al., 2015; Mustafi et al., 2018) or Lewis lung carcinoma cells (Campbell et al., 2015) implanted into mice grow more quickly in *Gulo^−/−^* mice partially depleted of dietary ascorbate than in wild-type mice. Hypoxia-inducible factor (HIF) levels increase in ascorbate-depleted cells, likely because HIF degradation is ascorbate dependent (Campbell et al., 2015). However, Lewis lung carcinoma cells grow more slowly in *Gulo^−/−^* mice completely depleted of dietary ascorbate, possibly due to angiogenesis defects (Telang et al., 2007). Ascorbate depletion had minimal to slightly inhibitory effects on premalignant gastric lesions induced by *Helicobacter pylori* infection (Lee et al., 2008) and inhibited the growth of fibrosarcoma cells implanted in guinea pigs with scurvy (Robertson et al., 1949). Thus, whether ascorbate promotes or inhibits cancer development depends on its plasma levels, the cancer type and whether experiments used cell lines adapted to ascorbate-free culture conditions. It has been proposed that ascorbate suppresses cancer development non-cell autonomously by modulating the production or function of specific immune cell types (Cameron et al., 1979; Magri et al., 2020) or the levels of collagen or hyaluronan (Cameron and Pauling, 1974). Experiments to directly test these ideas by depleting ascorbate in specific immune or extracellular-matrix-producing cells in a cancer context have not been performed. It has not been tested *in vivo* whether ascorbate regulates the development of any cancer by acting cell-autonomously in cancer-initiating cells. Thus, despite the long history of work on ascorbate and cancer, the cellular and molecular targets of ascorbate in the development of most cancers have not been defined with rigorous loss-of-function experiments. This is essential in order to understand whether ascorbate has physiological functions in any cancer and whether its repletion in ascorbate-depleted individuals can help prevent or treat cancer.

*In vitro*, ascorbate can kill many cancer cells (Shenoy et al., 2018) including leukemia cells (Brabson et al., 2023; Cimmino et al., 2017; Long et al., 2022; Huang et al., 2020; Liu et al., 2016; Noguera et al., 2017; Mingay et al., 2018). Most of these effects reflect non-specific reactive oxygen species (ROS) generation (Padayatty and Levine, 2016; Halliwell, 2014; Chen et al., 2005; Schoenfeld et al., 2017; Jankowski and Rabinowitz, 2022), but in some cases its activity is more specific*.* For example, ascorbate inhibits chemically induced transformation of an embryonic fibroblast cell line at low doses without killing the cells, suggesting that it does so by acting on a specific enzyme rather than via ROS generation (Benedict et al., 1980). Ascorbate kills myeloid leukemia cells *in vitro* by stimulating TET activity and reactivating endogenous retroviruses (Liu et al., 2016). Similarly, ascorbate suppresses *Tet2-*mutant HSC serial colony formation by promoting TET2/3 activity rather than via extracellular ROS generation (Cimmino et al., 2017).

### Supraphysiological ascorbate and cancer treatment in experimental models

**Fig. 1.**
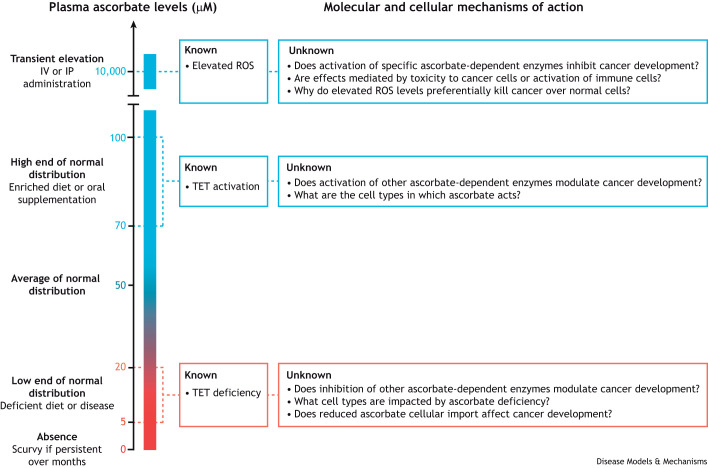
**Physiological and pharmacological ascorbate plasma concentrations in humans and their proposed mechanisms of action in cancer modulation.** Supraphysiological concentrations achieved through IV administration elevate ROS and kill cancer cells preferentially over normal cells via mechanisms that remain unclear. High physiological ascorbate concentrations achieved through diet or oral supplementation stimulate activity of TET leukemia suppressor enzymes in hematopoietic cells. Ascorbate deficiency leads to TET deficiency and promotes leukemia development. The cell types targeted by ascorbate and whether there are additional molecular mechanisms of action in most cancers remain unclear. IP, intraperitoneal; IV, intravenous; ROS, reactive oxygen species; TET, ten-eleven translocation.

Intravenous ascorbate administration elevates plasma ascorbate concentrations from ∼50 μM to ∼10-20 mM in both humans and mice, a ∼200-fold spike that returns to normal within a few hours (Fig. 1) (Chen et al., 2007; Yun et al., 2015). Many cancers in mice, including glioblastoma (Chen et al., 2008; Schoenfeld et al., 2017; Jankowski and Rabinowitz, 2022), non-small cell lung cancer (Schoenfeld et al., 2017), lymphoma (Luchtel et al., 2020), myelodysplastic syndrome (Huang et al., 2020), AML (Brabson et al., 2023; Cimmino et al., 2017; Long et al., 2022), colorectal cancer (Yun et al., 2015; Di Tano et al., 2020), melanoma (Magri et al., 2020; Xu et al., 2019), breast cancer (Magri et al., 2020), ovarian cancer (Ma et al., 2014), pancreatic cancer (Du et al., 2015, 2010; Espey et al., 2011), kidney cancer (Ge et al., 2018), liver cancer (Lv et al., 2024) and bladder cancer (Peng et al., 2018) respond to these very high, termed ‘pharmacological’ ascorbate doses. For example, pharmacological ascorbate inhibits AML growth in combination with poly (ADP-ribose) polymerase (PARP) inhibitors, which target DNA repair pathways (Cimmino et al., 2017; Brabson et al., 2023). In some cases, the effectiveness of pharmacological ascorbate requires the immune system, including cytotoxic T cells, or immunotherapy (Magri et al., 2020; Xu et al., 2019; Luchtel et al., 2020; Lv et al., 2024), but, in other cases, ascorbate is effective against xenografted tumors in immunocompromised mice (Chen et al., 2008; Schoenfeld et al., 2017; Yun et al., 2015; Ge et al., 2018; Jankowski and Rabinowitz, 2022). The precise mechanisms by which pharmacological ascorbate kills cancer cells are a subject of debate (Jankowski and Rabinowitz, 2022; Yun et al., 2015; Chen et al., 2007; Schoenfeld et al., 2017). The most likely mechanism involves extracellular generation of hydrogen peroxide, which inflicts cellular damage such as oxidative stress, NAD^+^ depletion and DNA damage, effects opposite to the normal antioxidant role of ascorbate (Padayatty and Levine, 2016; Halliwell, 2014; Chen et al., 2005; Schoenfeld et al., 2017; Jankowski and Rabinowitz, 2022). The toxic effects of pharmacological ascorbate are enhanced by copper (Bram et al., 1980; Stich et al., 1976) and iron (Jankowski and Rabinowitz, 2022; Schoenfeld et al., 2017) and counteracted by selenium (Jankowski and Rabinowitz, 2022). Most experiments to dissect pharmacological ascorbate cytotoxicity mechanisms were done *in vitro*, and it is unclear whether the same mechanisms apply *in vivo*. The mechanisms that render cancer cells more sensitive than normal proliferating cells to this treatment are unclear. The fact that ascorbate levels increase for just a few hours after intravenous administration may limit the clinical utility of this approach to specific cancers that are highly sensitive to oxidative stress.

### The role of ascorbate in human cancer

#### Association of low ascorbate levels with cancer

Plasma ascorbate concentrations in European and North American populations vary considerably from ∼5 to 100 μM, with an average of ∼50 μM (Fig. 1) (Khaw et al., 2001; Loria et al., 2000; Schleicher et al., 2009). This variation is higher than in species that can synthesize ascorbate; for example, plasma concentrations in mice are ∼30-90 μM (Chen et al., 2007; Agathocleous et al., 2017). Ascorbate levels fall in patients with blood cancer (Kyhos et al., 1945; Cuttle, 1938; Waldo and Zipf, 1955; Ottone et al., 2022; Premnath et al., 2023; Liu et al., 2016; Huijskens et al., 2016; Mangione et al., 2024), smokers and populations deprived of fresh food, or following surgery, sepsis or inflammation (Long et al., 2003; Marcus et al., 1991; Du et al., 2003; Chevion et al., 1999; Fowler et al., 2014; Galley et al., 1996; Crandon et al., 1958; Li et al., 2023). Less comprehensive data from African, Latin American or South Asian countries show average plasma ascorbate levels of ∼10-30 μM, suggesting that a high fraction of the global population is ascorbate deficient (Rowe and Carr, 2020). Low ascorbate levels are associated with increased mortality from cancer (Khaw et al., 2001; Loria et al., 2000; Goyal et al., 2013; Eichholzer et al., 1996; Wang et al., 2018). Genetic variation accounts for only a small proportion of variation in circulating ascorbate levels (Zheng et al., 2021; Timpson et al., 2010), making it challenging to use human genetics to test a causal relationship.

It will be important to understand the mechanisms by which intravenous ascorbate kills cancer cells […] to target this treatment most effectively.

#### Clinical trials in humans

Interventional trials showed that ascorbate supplementation does not prevent cancer in healthy humans (Frei et al., 2012). However, these trials were not performed in individuals with low ascorbate levels at baseline (Frei et al., 2012). For example, supplementation trials of individuals at high risk for development of cancer or cardiovascular disease elevated plasma ascorbate from 43-48 μM to 59-62 μM (Heart Protection Study Collaborative Group, 2002; Plummer et al., 2007), values well within the interquartile range in the human population (Khaw et al., 2001; Loria et al., 2000; Schleicher et al., 2009) and therefore unlikely to reflect the impact of low or high ascorbate levels. In large prevention trials in high socioeconomic status individuals (Sesso et al., 2008), ascorbate in the intervention group was likely elevated by an even more marginal amount (Levine et al., 2011; Padayatty and Levine, 2009; Lykkesfeldt and Poulsen, 2010). Thus, even if ascorbate was involved in cancer development, no effects on cancer incidence would be expected in these trials. Most trials of ascorbate as cancer treatment have been conducted in solid cancers, for which there has been no evidence of a role for ascorbate, nor an understanding of a possible mechanism of action. For example, the landmark Mayo clinic trials, which showed that mega-doses of oral ascorbate were ineffective in cancer, supplemented patients who had not been pre-selected for deficiency and had solid cancers (Creagan et al., 1979; Moertel et al., 1985). Patients with leukemia were excluded (Creagan et al., 1979; Moertel et al., 1985). In contrast, initial data described in conference proceedings from a randomized clinical trial in patients with early-stage myeloid neoplasms and below-average baseline ascorbate levels showed that oral ascorbate supplementation substantially elevated plasma ascorbate and improved survival (Mikkelsen et al., 2024).

The many papers showing that intravenous (pharmacological) ascorbate can inhibit cancer growth in mouse models (Chen et al., 2008; Schoenfeld et al., 2017; Jankowski and Rabinowitz, 2022; Yun et al., 2015; Di Tano et al., 2020; Magri et al., 2020; Xu et al., 2019; Ma et al., 2014; Du et al., 2015, 2010; Espey et al., 2011; Ge et al., 2018; Luchtel et al., 2020; Lv et al., 2024; Huang et al., 2020; Brabson et al., 2023; Cimmino et al., 2017; Long et al., 2022; Peng et al., 2018) stimulated ongoing trials in several cancers (Furqan et al., 2022; Wang et al., 2022; Shenoy et al., 2018). Recent small phase 2 randomized trials showed that intravenous ascorbate as an adjunct to chemotherapy extended survival from 8 to 16 months in patients with stage IV pancreatic cancer (Bodeker et al., 2024) but did not benefit patients with metastatic prostate cancer (Paller et al., 2024). In a randomized trial of patients with AML treated with decitabine, an inhibitor of DNA methylation, intravenous ascorbate extended median survival from 9 to 15 months (Zhao et al., 2018). Initial data from another trial showed limited or no clinical efficacy of intravenous ascorbate in patients with myeloid neoplasm (Xie et al., 2024). These trials treated small numbers of patients, and larger trials are needed for definitive results. Intravenous administration elevates ascorbate only transiently, its administration is limited to the clinic, and its mechanism of action and selectivity for cancer cells are not well understood. It could have an anti-cancer effect if given frequently, perhaps in combination with increased iron or copper or depleted selenium to enhance its toxic effects (Bram et al., 1980; Stich et al., 1976; Jankowski and Rabinowitz, 2022; Schoenfeld et al., 2017). It will be important to understand the mechanisms by which intravenous ascorbate kills cancer cells *in vivo*, and the mechanisms that determine selectivity for cancer cells over normal cells, in order to target this treatment most effectively.

It has been suggested that ascorbate could be used as a precision medicine tool in *TET2-*mutant myeloid leukemia (Cimmino et al., 2018; Xie et al., 2024). However, it must be emphasized that ascorbate administration increases ascorbate levels in a sustained manner only in ascorbate-deficient individuals but not in individuals without ascorbate deficiency (Frei et al., 2012). Ascorbate deficiency causes a TET-deficient state in blood or hematopoietic cells that can be reversed by ascorbate supplementation (Agathocleous et al., 2017; Gillberg et al., 2019; Taira et al., 2023). TET deficiency collaborates with many other leukemogenic mutations (Shih et al., 2015; Zhang et al., 2016). Therefore, I predict that ascorbate supplementation to patients with myeloid neoplasms will most benefit those who, at baseline, have below-average ascorbate levels, irrespective of whether their leukemias carry *TET2* mutations. It has also been suggested that ascorbate administration suppresses *TET2*-mutant clonal hematopoiesis (Cimmino et al., 2018). However, ascorbate administration, even when it causes a sustained increase in ascorbate levels, should increase TET activity in both mutant and wild-type HSCs. Thus, it will not necessarily reduce the clonal competitiveness of *TET2*-mutant over wild-type HSCs. This is consistent with experiments in mice that suggested that ascorbate does not reduce the clonal competitiveness of *Tet2*-deficient HSCs (Agathocleous et al., 2017; Nakauchi et al., 2022). I predict that ascorbate administration will benefit ascorbate-deficient individuals with clonal hematopoiesis, not by changing clonal competition but by preventing a TET-deficient state that collaborates with clonal hematopoiesis mutations (in *TET2* or other genes) to cause leukemia or exacerbate inflammation. Early data from a clinical trial in early-stage myeloid neoplasms support this idea (Mikkelsen et al., 2024).

## Conclusions

It was necessary to use the naturally *Gulo*-deficient guinea pig as a model system to establish, after centuries of debate, that scurvy is a nutritional deficiency (Holst and Frolich, 1907) and then to discover vitamin C (Svirbely and Szent-Gyorgyi, 1932). Likewise, I argue that it is now necessary to use genetic models of ascorbate depletion, such as *Gulo*- and *Slc23a1/a2*-knockout mice, to understand the role of ascorbate in physiology and disease (Agathocleous et al., 2017; Thaler et al., 2022; DiTroia et al., 2019; Sotiriou et al., 2002; Bornstein et al., 2003; Maeda et al., 2000; Corpe et al., 2010). In addition to myeloid neoplasms, good candidates to test the physiological role of ascorbate are other cancers in which TET activity is reduced (Rasmussen and Helin, 2016). Underlying mechanisms must be established in the same way as for any other endogenous molecule, i.e. using rigorous cell-type-specific loss-of-function experiments. Myeloid neoplasms are the only context for which physiological ascorbate levels have been shown to promote the activity of a tumor suppressor (Agathocleous et al., 2017; Gillberg et al., 2019; Taira et al., 2023) and to increase survival *in vivo*, in mice and perhaps in humans (Agathocleous et al., 2017; Mikkelsen et al., 2024). Ascorbate deficiency is common in patients with leukemia for unknown reasons (Kyhos et al., 1945; Cuttle, 1938; Waldo and Zipf, 1955; Ottone et al., 2022; Premnath et al., 2023; Liu et al., 2016; Huijskens et al., 2016) and is also common in many unaffected individuals. Clinical trials focusing on ascorbate-deficient individuals whose ascorbate concentrations can be persistently and substantially elevated by oral supplementation, or a combination of intravenous and oral supplementation, will test whether ascorbate can prevent or slow progression of human leukemia. If that is the case, it would suggest that patients with leukemia, or those at risk of developing leukemia, must avoid becoming ascorbate deficient.
